# Proteomics insights: exploring the inflammatory and metabolic signatures of ethnicity and change in physical activity in non-diabetic hyperglycaemia

**DOI:** 10.1016/j.ebiom.2025.106006

**Published:** 2025-11-03

**Authors:** Joseph Henson, Amr Ghit, Aikaterina Tziannou, Emily James, Charlotte L. Edwardson, Nicolette C. Bishop, Cameron Razieh, Donald J.L. Jones, Thong Huy Cao, Melanie J. Davies, Kamlesh Khunti, Thomas Yates

**Affiliations:** a Diabetes Research Centre, College of Life Sciences, University of Leicester, Leicester, UK; b National Institute for Health and Care Research Leicester Biomedical Research Centre, Leicester, UK; c Department of Medicine and Aging Sciences, “G. d’Annunzio” University of Chieti-Pescara, Chieti, Italy; d Center for Advanced Studies and Technology (CAST), “G. d’Annunzio” University of Chieti-Pescara, Chieti, Italy; e National Centre for Sport and Exercise Medicine, School of Sport, Exercise and Health Sciences, Loughborough University, Loughborough, UK; f Leicester Real World Evidence Unit, Diabetes Research Centre, University of Leicester, Leicester, UK; g Leicester Cancer Research Centre, University Hospitals of Leicester NHS Trust, Leicester Royal Infirmary, Leicester, UK; h Leicester van Geest Multi-OMICS Facility, University of Leicester, Leicester, UK; i Department of Cardiovascular Sciences, College of Life Sciences, University of Leicester, Leicester, UK; j Leicester Diabetes Centre, University Hospitals of Leicester NHS Trust, Leicester, UK; k NIHR Applied Research Collaboration, East Midlands, UK

**Keywords:** Ethnicity, Inflammation, Metabolism, Physical activity, Proteomics

## Abstract

**Background:**

People of South Asian (SA) origin have an elevated risk of cardiometabolic dysfunction and respond differentially to physical activity. Mechanisms underpinning these observations are incompletely understood. We investigated protein signatures of ethnicity, changing physical activity and their interaction.

**Methods:**

A discovery analysis was conducted using data from 794 SA to 49,153 white European (WE) participants in UK Biobank, assessing associations between 726 plasma proteins and physical activity and ethnicity. Findings were validated in a targeted cohort of 58 individuals with non-diabetic hyperglycaemia, taken from a larger randomised controlled trial (PROPELS, n = 1366), stratified by ethnicity and changes in accelerometer assessed physical activity (steps/day) over 48 months. Individuals matched for ethnicity, age and sex that increased and decreased their physical activity over time were sampled, creating four groups (SA increasers [n = 15], SA decreasers [n = 15], WE increasers [n = 14], WE decreasers [n = 14]). Proteins were analysed using Olink inflammation and cardiovascular panels.

**Findings:**

In the discovery phase, 464 proteins were associated with physical activity and 375 with ethnicity. In the validation cohort, only interleukin-6 (IL-6) was significantly associated with physical activity, showing downregulation in those who increased activity. Sixteen proteins were validated for ethnicity, including upregulated chemokines (e.g., CCL28, CCL15, CCL13, CCL11, CCL26), hepatokines (FABP1), adipokines (FABP2), and pancreatic proteins (AMY2A, AMY2B), with CXCL14, GHRL and PRTN3 downregulated in SAs.

**Interpretation:**

Distinct proteomic profiles were associated with ethnicity and physical activity. IL-6 emerged as a key marker of physical activity response, while ethnicity-related proteins highlighted immune, inflammatory, and metabolic pathways.

**Funding:**

10.13039/501100020013NIHR Leicester Biomedical Research Centre.

**Research in context**

**Evidence before this study**

We reviewed existing literature (PubMed search from database inception to January 2025), using search terms related to Type 2 diabetes (T2DM) and non-diabetic hyperglycaemia, ethnicity, physical activity and plasma proteins. Non-diabetic hyperglycaemia is an important stage because it offers a chance to intervene before T2DM develops. South Asians (SA), the largest ethnic minority group in the UK, develop T2DM more frequently, more quickly and at a younger age and lower body weight than white Europeans (WE). One factor contributing to this higher risk is lower levels of physical activity among SA individuals. Additionally, new research shows that SAs and WEs respond differently to physical activity, with enhanced responses to insulin sensitivity but blunted responses to fat mass and body composition. Although there are growing numbers of studies investigating the proteomics of ethnicity, there is a lack of data in those with non-diabetic hyperglycaemia and no previous studies have investigated the protein signatures of changing physical activity, ethnicity and their interaction.

**Added value of this study**

This study adds new insight into potential pathways and mechanisms contributing to ethnic health and responses to changing physical activity, although no interactions between the two were validated.

**Implications of all the available evidence**

Although it is established that SA ethnicities have a higher risk of cardiometabolic disease, with recent data also showing health differences in response to physical activity, the mechanisms underpinning these observations have not been fully elucidated. Proteomics is adding new insight into the mechanisms underpinning ethnic health. This study highlights novel targets that could add new insight into the mechanisms and pathways underpinning SA health.

## Introduction

In 2021, the global prevalence of Type 2 diabetes mellitus (T2DM) was estimated to exceed half a billion (536.6 million), representing 10.5% of the total adult population aged 20–79 years.^1^ T2DM resides at one end of a continuous glucose control spectrum, with normal glucose levels at the other. In between, there exists a “high-risk” condition called prediabetes or non-diabetic hyperglycemia (NDH), prevalent in ∼542 million individuals worldwide.^1^ This intermediate stage of hyperglycaemia is important; it provides a potential window of opportunity to identify individuals at the greatest risk of developing T2DM to implement appropriate therapeutic strategies and has been the focus of diabetes prevention programmes.^2^ This is particularly pertinent for South Asian (SA) individuals, the largest ethnic minority group within the United Kingdom (UK). SAs have a two-to four-fold higher risk of developing T2DM and accelerated progression from NDH to T2DM, often occurring at a younger age and lower body mass index (BMI) compared to white European (WE) individuals.^3^, ^4^, ^5^

The reasons for this elevated risk are not fully understood. Physical activity participation, a cornerstone of current diabetes prevention and treatment guidelines in the UK, has been suggested as one of the reasons for the disparity in chronic disease risk.^6^ In a cohort at high risk of T2DM, SAs engaged in lower levels of moderate-to-vigorous physical activity and took fewer steps/day compared to WEs.^7^ Moreover, there are emerging findings of ethnicity-specific differences in response to physical activity or exercise training.^8^, ^9^, ^10^ For instance, changes in body composition and fat metabolism are less pronounced in SAs (vs WEs),^8^ whereas changes to glucose metabolism and insulin sensitivity were found to be more pronounced in SAs in response to physical activity.^9^^,^^10^ To implement effective lifestyle interventions, specific physiological pathways and disparate responses to physical activity need further elucidation.

Proteomic analyses are increasingly used to identify novel markers of disease to optimise personalised medicine. Although such technologies have been harnessed to explore the therapeutic and dynamic responses to physical activity,^11^^,^^12^ none have explored whether the results are moderated by ethnicity. This approach has the potential to identify novel proteins and biological pathways that regulate changes in physical activity, thus enabling new insights into the risk of metabolic dysfunction in high-risk ethnic groups. This study initially explores the plasma proteome of WE and SA adults to: identify novel proteins and examine how they are associated with physical activity and whether this association is modified by ethnicity (discovery); with a primary aim of then validating these findings by examining how these proteins change in response to physical activity in an independent cohort of WE and SA adults with NDH.

## Methods

### Discovery proteomic analysis

#### Cohort definition

Plasma samples collected as part of the UK Biobank cohort were utilised for the discovery analysis. In the UK Biobank Pharma Proteomics Project, 726 proteins covering inflammation and cardiovascular pathways were available in 54,239 participants, which were randomly selected and broadly representative of the wider Biobank cohort (n = 502,414). Of these, 794 SA and 49,153 WE participants had data corresponding to exposures (leisure time physical activity (MET-minutes/week), analysed as reported previously,^13^ ethnicity and covariates of interest (age, sex, social deprivation (Townsend deprivation index), smoking status (current/former/never), cancer (yes/no), diabetes (yes/no), cardiovascular disease(s) (yes/no) see Table 1 . Analyses were conducted using project number 33266.

**Table 1:** Participant characteristics of the UK Biobank discovery cohort.

	n = 49,947
White European (%)	49,153 (98.4)
South Asian (%)	794 (1.6)
Leisure time physical activity (MET-minutes/week)	519.6 [154.8, 1183.2]
Female (%)	26,898 (53.9)
Male (%)	23,049 (46.1)
Age (years)	59 [51, 64]
Smoking status (Current, %)	5227 (10.5)
Townsend deprivation index	−2.2 [−3.7, 0.5]
BMI (kg/m^2^)	26.8 [24.2, 29.9]
Diabetes (yes)	2603 (5.2)
Cancer (yes)	4318 (8.6)
Cardiovascular disease(s) (yes)	3742 (7.5)

Data are reported as median [interquartile range] or number (%).

#### Proteomic analysis

The plasma samples were measured at Olink's facilities in Uppsala Sweden, using the antibody-based Olink Explore 3072 PEA. In brief, the target protein binds to the double oligonucleotide-labelled antibody probe with high specificity, and then the microfluidic real-time PCR amplification of the oligonucleotide sequence is used to quantitatively detect the resulting DNA sequence. Using internal and external controls, the resulting threshold cycle (Ct)-data were processed for quality control and normalised. Normalised Protein expression (NPX) values were provided as the final assay read-out, which was an arbitrary log2-scale unit corresponding to higher protein levels. Detailed information on the proteomics technology as well as the normalisation and quality control steps has already been published^14^

### Proteomic analysis used for validation

#### Cohort definition

Participants were recruited by purposeful sampling as part of a larger randomised controlled trial (The Promotion Of Physical activity through structured Education with differing Levels of ongoing Support for those with pre-diabetes: PROPELS), which has been described in detail elsewhere.^15^^,^^16^ Briefly, this was a three-arm (control, Walking Away, Walking Away plus), parallel-group, randomised controlled trial (n = 1366), conducted over 48 months. Measurements occurred at baseline, 12 months and 48 months. Those in the control arm were provided with an information leaflet targeting knowledge and perceptions of diabetes risk and the importance of physical activity. The Walking Away arm consisted of a 3-h, group-based behavioural intervention aimed at targeting knowledge and perceptions of risk factors for T2DM, outcome expectations around the effectiveness of physical activity at managing those risk factors, physical activity self-efficacy, and provisions of pedometers to support goal setting and self-monitoring. The Walking Away Plus arm included the standard Walking Away programme, plus annual refresher sessions and additional follow-on support in the form of a tailored mHealth intervention.

### Participants

Eligible participants were recruited from primary care, using records held at their individual GP (General Practitioner) practice. In the first instance, participants were identified using data collected by the NHS Health Checks programme, a screening programme run in the UK designed to identify and treat vascular disease risk (heart disease, stroke, diabetes, and kidney disease) in adults aged 40–74 years. An automated search algorithm was used to identify those with a blood glucose or HbA1c value indicating NDH within the preceding 5 years and who were not currently enrolled in other diabetes prevention programmes. NDH was defined as fasting glucose ≥5.5 mmol/l and <7.0 mmol/l, 2-h post–challenge glucose ≥7.8 mmol/l and <11.1 mmol/l or HbA1c ≥ 6.0% (42 mmol/mol) and <6.5% (48 mmol/mol). To be considered eligible, participants had to be aged 40–74 years (25–74 years if SA), with access to a mobile phone. Participants were excluded if they were unable to undertake walking activity, were pregnant or unable to provide informed consent.

### Ethics

Informed consent was provided by all eligible participants. Both studies gained full ethical and governance approvals. For PROPELS, ethical approval was granted by the NHS National Research Ethics Service, East-Midlands Leicester Committee (Ethics number: 12/EM/0151). The trial was registered at ISRCTN83465245. UK Biobank received research ethics approval from the North West Multi-Centre Research Ethics Committee as a Research Tissue Bank in 2011 (Ethics number: 21/NW/0157).

### Ambulatory activity

Participants wore an accelerometer (Actigraph GT3X+, Florida, USA) around their waist, for seven consecutive days during waking hours. Data were collected at 100Hz and reintegrated into 60-s epoch files for analysis purposes. Non-wear time was defined as a minimum of 60 min of continuous zero counts; days with at least 600 min of wear time were considered valid. To be included in the analysis, participants required a minimum of any three valid days. A commercially available data analysis tool (KineSoft version 3.3.76, Kinesoft, Loughborough, UK; www.kinesoft.org) was used to process the accelerometer data.

### Cardio-metabolic, anthropometric and demographic variables

Venous blood samples were collected following an overnight fast, with plasma obtained by centrifugation (3000 rpm for 15 min at 4 °C) and stored at −80 °C until laboratory analysis.

Body weight and height were measured to the nearest 0.1 kg and 0.1 cm, respectively. BMI was calculated to the nearest 0.1 kg/m^2^. Information on ethnicity was obtained following an interview administered protocol. Participants were defined as WE if they self-reported to be White British, White Irish or any other white background, while SA was defined when self-reporting to be Indian (under the category of Asian or Asian British). An Index of Multiple Deprivation (IMD) score was assigned to the participant's resident area to indicate socioeconomic deprivation status. IMD scores are publicly available, continuous measures of compound social and material deprivation, calculated using a variety of data including current income, employment, education and housing.

To validate the discovery proteomic findings, 58 individuals from the PROPELS study were purposefully selected from the wider available PROPELS cohort (n = 1366) and placed into four distinct groups, dichotomised by ethnicity (WE or SA) and changes in physical activity (increase or decrease) across the 48-month period as: (1) WE-increasers, (2) WE-decreasers, (3) SA-increasers, and (4) SA-decreasers. Physical activity categories were selected based on ranking those that had the largest increase or decrease in ambulatory activity (steps per day) at 48 months. From these study specific rankings, we selected individuals in the top and bottom two deciles. To ensure consistency in behaviour change, we further required that the direction of change (increasing or decreasing) was evident at the 12-month time point. To reduce heterogeneity amongst subgroups, these groups were also matched for sex and age.

#### Proteomic analysis

The corresponding plasma markers obtained at baseline, 12 months and 48 months were assessed using the commercially available Olink® Target 96 Inflammation and Cardiovascular panels (Thermo Fisher Scientific, Massachusetts, US). These were facilitated through Explore proximity extension assay (PEA) technology^17^ and carried out in a UK certified laboratory (University of Oxford). Of note, the same Inflammation and Cardiovascular panels used in the discovery phase were available and followed the same methods as detailed above. Processed data are not publicly available but can be requested from the corresponding author and used for collaborative research purposes.

### Statistics

For the discovery phase of the analysis, we conducted a series of linear regression models to evaluate the impact of leisure time physical activity (MET-minutes/week) and ethnicity (SA vs WE) on each protein (n = 726). Models assessed the main effects of physical activity, ethnicity and their interaction, within a single model, alongside covariates including age, sex, social deprivation (Townsend deprivation index, with higher values indicating a greater deprivation), smoking status (current/former/never), cancer (yes/no), diabetes (yes/no) and cardiovascular disease(s) (yes/no) included as covariates. To account for multiple comparisons and reduce the likelihood of Type I errors in the discovery phase, we applied a Bonferroni correction. This method adjusts the significance threshold by dividing the conventional alpha level (i.e., 0.05) by the number of comparisons made (i.e., 726), thereby providing a more stringent criterion for statistical significance. For each unique protein, beta coefficients, *p*-values, and 95% CIs representing normalised protein levels per unit change in physical activity and ethnicity (SA vs WE) were extracted for each protein, with significant proteins carried forward to the validation stage (Supplementary File 1). The models were fitted using the lme function from the nlme package in R (version 4.3.1). The assumptions required for linear regression were evaluated on a subset of models (n = 50) which were randomly selected from the full set of proteins. This random selection was designed to yield a representative sample of the modelling framework applied across all proteins. The confirmation that assumptions were met, together with the principles of the Central Limit Theorem,^18^ supports the inference that these assumptions are likely to hold for the broader set of models, particularly in light of the homogeneity in predictor and biomarker distributions.

For validation, proteins identified in the discovery phase were selected for further analysis. A mixed-effects linear model was undertaken, which included the fixed effects of ambulatory activity (increase vs decrease), ethnicity (SA vs WE), time as a continuous variable as well as the interactions between physical activity and time and ethnicity and time. Baseline covariates for randomisation sequence (control, Walking Away, Walking Away plus), age, sex, baseline steps (per day) and protein expression, were also added to the models. A random intercept for each participant was included to account for within-subject variability. Repeated measures were analysed using an exchangeable correlation matrix. The main terms reported in this analysis are physical activity category by time interaction (assessing whether increasing vs decreasing physical activity was associated with changes to the proteome), ethnicity (assessing whether on average there was a difference in the proteome between ethnicities) and the ethnicity by time interaction (assessing whether differences between ethnicities changed over time). An additional model also included a triple interaction (ethnicity by physical activity by time). Finally, all models were further adjusted for BMI to assess whether associations were independent of adiposity. For ease of interpretation, results were scaled and presented as the normalised protein change per 12 months (in increasers vs decreasers in physical activity and in SAs vs WEs). Given the relatively small sample size and limited power of the validation cohort, we employed the Benjamini-Hochberg procedure to control the false discovery rate (FDR) at 0.1 for validation.

### Bioinformatics

The R package “Olink®Analyze” was used to identify and extract available proteins, visualised using forest, volcano, and dot plots generated using standard R package “ggplots2”. For those proteins that differed significantly between ethnicity and/or physical activity following the validation stage, Gene Ontology (GO) enrichment analysis (https://geneontology.org/) and pathway analyses (R Package: ClustProfiler) using the Kyoto Encyclopaedia of Genes and Genomes (KEGG) were undertaken (Supplementary Files 2 and 3). The Protein–Protein Interaction (PPI) network model was constructed using data from the STRING database (https://string-db.org/). Cytoscape software (version 3.10.1) was used on the PPI network to identify hub genes.

### Role of funders

The funder had no role in the collection, analysis, and interpretation of data; in the writing of the report; or in the decision to submit the paper for publication.

## Results

### Discovery within UK biobank

**Supplementary Figure S1:**
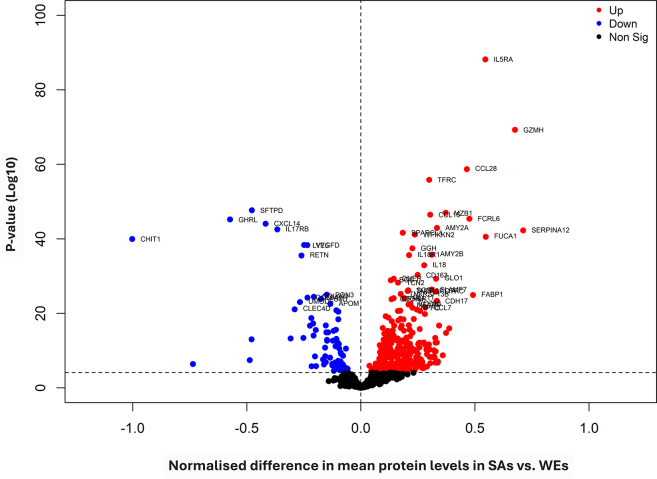
**Volcano plot of ethnicity -associated proteins in UK Biobank**. Values are adjusted for age, sex, social deprivation smoking status, cancer, cardiovascular disease and diabetes; values reflect the normalised difference in protein levels in SAs *vs.* WEs. P-values are nominal and have not been corrected for multiple comparisons.

Table 1 includes the participant characteristics for the discovery cohort, where a total of 726 plasma proteins were included in the initial analysis (see Supplementary File 1 for the complete list of proteins and corresponding model results). In analyses examining the association between ethnicity and circulating plasma protein levels, a total of 375 proteins were identified after applying a stringent Bonferroni correction. Of these, 298 (79.5%) showed stronger positive associations (upregulated) in individuals of SA ethnicity compared to WEs. In contrast, only 77 proteins (20.5%) were downregulated in SAs (Supplementary Figure S1).

**Supplementary Figure S2:**
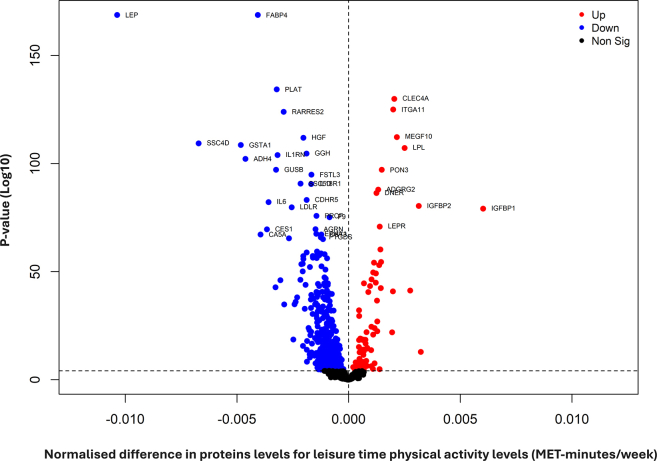
**Volcano plot of physical activity-associated proteins in UK Biobank**. Values are adjusted for age, sex, social deprivation smoking status, cancer, cardiovascular disease and diabetes; values reflect the the normalised difference in proteins levels for leisure time physical activity levels (MET-minutes/week). P-values are nominal and have not been corrected for multiple comparisons.

In comparison, 464 proteins showed associations with leisure time physical activity and were therefore carried forward to the validation stage. Among these, the majority (n = 388; 83.6%) were downregulated with high physical activity. The remaining proteins were upregulated, although these tended to be weaker in magnitude (Supplementary Figure S2).

A single protein (SERPINA12) changed differentially between physical activity and ethnicities, being upregulated in SAs (Supplementary File 1).

### Validation within propels

Participant characteristics, stratified by physical activity and ethnicity criteria, are displayed in Table 2. The SA and WE groups were well matched for baseline and change in physical activity levels across the increasers and decreasers groups (categories within 500 steps/day).

**Table 2:** Participant characteristics.

	South Asian		White European	
	Increase (n = 15)	Decrease (n = 15)	Increase (n = 14)	Decrease (n = 14)
Female (n, %)	7 (46.7)	7 (46.7)	7 (50.0)	7 (50.0)
Male (n, %)	8 (53.3)	8 (53.3)	7 (50.0)	7 (50.0)
Age (years)	55.4 ± 8.7	52.9 ± 9.9	58.2 ± 8.0	54.4 ± 8.8
IMD (Decile)	5 [3]	4 [6]	6.5 [5]	6 [3.25]
Smoking status				
Never smoked (n, %)	12 (80.0)	12 (80.0)	10 (71.4)	6 (42.9)
Previous smoker (n, %)	1 (6.7)	3 (20.0)	4 (28.6)	6 (42.9)
Current smoker (n, %)	2 (13.3)	0 (0.0)	0 (0.0)	2 (14.2)
HbA1c (mmol/mol)	41 ± 3	38 ± 2	39 ± 4	39 ± 4
HbA1c (%)	5.9 ± 0.3	5.6 ± 0.2	5.7 ± 0.3	5.7 ± 0.3
BMI (kg/m^2^)	30.2 ± 11.2	26.8 ± 4.7	27.3 ± 5.0	29.8 ± 3.6
Baseline steps per day	7077 ± 3053	8195 ± 2063	6643 ± 2123	8630 ± 1678
Change in steps at 12 months	614 ± 2254	−556 ± 1412	387 ± 1025	−874 ± 1182
Change in steps at 48 months	2750 ± 1390	−2955 ± 1294	2391 ± 729	−3433 ± 1089

Data presented as mean ± standard deviation, median [interquartile range] or number (column percentage).

IMD: Index of multiple deprivation.

BMI: Body mass index.

**Fig. 1:**
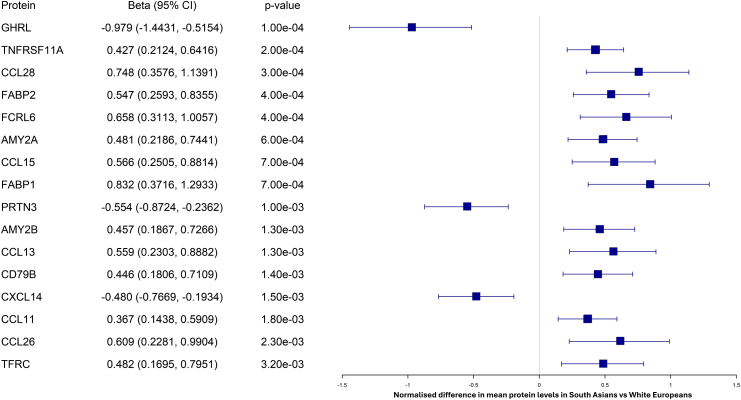
**Forest plot of ethnicity associated proteins in PROPELS**. Values are adjusted for age, sex, randomisation sequence (control, Walking Away, Walking Away plus), and baseline steps (per day). Values reflect the normalised difference in mean protein levels. *p*-Values are nominal and have not been corrected for multiple comparisons.

For ethnicity, a total of 16 proteins identified in the discovery stage were also significantly associated with ethnicity at validation and exhibited consistent directional effects with those observed in the discovery cohort, following correction for multiple testing (Fig. 1). Of these, 13 proteins were upregulated in SAs compared to WEs, and 3 proteins were downregulated (Fig. 1 and Supplementary Table S1). After accounting for multiple testing, no proteins passed validation for the ethnicity by time interaction, suggesting identified ethnic differences were stable over time (Supplementary File 2).

Of the physical activity proteins identified in the discovery phase, only interleukin-6 (IL-6) demonstrated a statistically significant association with physical activity in the validation cohort after applying the Benjamini-Hochberg procedure for multiple testing (Supplementary Table S2). Specifically, in the PROPELS cohort, IL-6 levels were significantly downregulated in participants who increased their physical activity over the 4-year follow-up period.

SERPINA12 was not validated within the physical activity by time by ethnicity interaction, meaning there was no evidence that ethnicity modified responses to changing physical activity for any of the investigated proteins.

### Bioinformatics analysis

#### Ethnicity related proteins

Ethnicity-associated protein pathway and GO enrichment analyses are presented in Supplementary File 3.

**Fig. 2:**
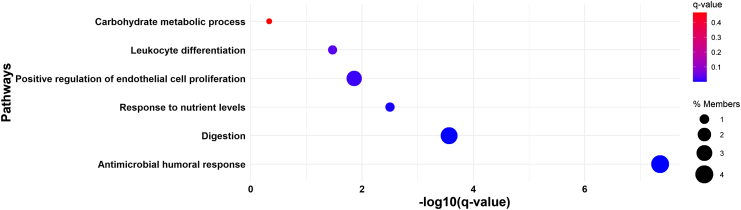
**Enriched KEGG pathways for dysregulated plasma proteins related to ethnicity in PROPELS**. Circle size represents the percentage of genes involved in each pathway (% Members), and circle colour reflects the q-value, with blue indicating higher significance and red indicating lower significance.

Key immune and inflammatory pathways identified included cytokine–cytokine receptor interaction, viral protein interaction with cytokine and cytokine receptor, and the chemokine signalling pathway. These were significantly enriched by chemokines such as CCL28, CCL15, CCL13, CXCL14, CCL11, and CCL26, the majority of which were upregulated in SA (Fig. 2).

In addition to immune pathways, several metabolic and digestive pathways were enriched. These included fat digestion and absorption and PPAR signalling, driven by FABP2 and FABP1, as well as carbohydrate metabolism pathways involving AMY2A and AMY2B – all of which were upregulated in SAs.

GO enrichment analysis further supported these findings, revealing strong overrepresentation of immune-related biological processes, particularly those involving humoural responses, chemotaxis, and leucocyte migration.

**Fig. 3:**
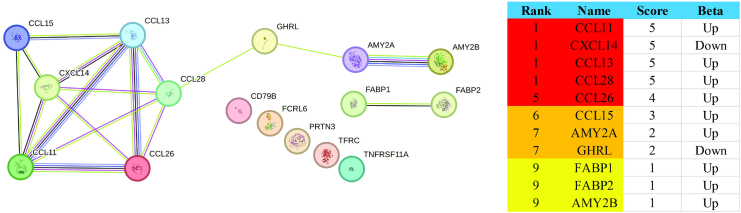
**Protein–protein interaction model for ethnicity -associated proteins in PROPELS**. Edges in the network indicate interactions between proteins, while nodes represent individual proteins. Edge colours: Known Interactions–Light Blue (Curated databases), Purple (Experimentally determined); Predicted Interactions–Green (Gene neighbourhood), Red (Gene fusions), Blue (Gene co-occurrence); Others–Black (Co-expression), Light Green (Text mining), Violet (Protein homology). On the right of the figure, genes are ranked based on their centrality in the network (the lower the rank, the higher the importance) with higher scores indicating greater relevance or connectivity. Colours represent the number of interacted proteins: Red (4–5), Orange (2–3), Yellow (1). The beta column denotes the direction of association, with ‘up’ indicating increased expression and ‘down’ indicating decreased expression.

Protein–protein interaction network analysis (Fig. 3) highlighted CXCL14 as a central node, interacting with multiple chemokines including CCL28, CCL15, CCL13 and CCL26, suggesting a coordinated chemokine-driven signalling axis.

#### Physical activity related proteins

As IL-6 was the sole significant protein, pathway and GO enrichment analyses were not conducted.

## Discussion

In the present study, we examined the associations of ethnicity (SA, WE) and change in physical activity on the plasma proteome. A total of 464 proteins were associated with leisure-time physical activity in UK Biobank and progressed to the validation stage, while 375 proteins were identified in relation to ethnicity and circulating plasma protein levels. Following validation, a total of 16 ethnicity-associated proteins and one physical activity-associated protein (IL-6) were identified, with no overlap between the two. Further, no proteins were validated for a physical activity by ethnicity interaction. Ethnicity-associated proteins include novel hepatokines, adipokines and pancreatic proteins, with enrichment in metabolic and digestive pathways, such as fat digestion, PPAR signalling and carbohydrate metabolism. These findings highlight distinct molecular signatures associated with ethnicity and physical activity, with IL-6 emerging as a central marker in response to physical activity, and a broader chemokine-driven immune network characterising ethnic differences.

The prominence of the association of IL-6 with changing physical activity aligns with its well-established role in the literature as a pleiotropic cytokine that bridges immune regulation, metabolic control and inflammatory responses.^19^ Recent studies have reinforced IL-6's critical function in modulating chronic inflammation, with the therapeutic inhibition of IL-6 signalling now a cornerstone in the management of several inflammatory diseases.^20^ Notably, IL-6 exhibits dual and sometimes opposing roles depending on its tissue of origin and mode of release. As a myokine, IL-6 is acutely released from skeletal muscle during physical activity, where it exerts anti-inflammatory and metabolic benefits, including enhanced glucose uptake and lipid oxidation. Conversely, as an adipokine, IL-6 is chronically elevated in obesity and NDH, contributing to low-grade systemic inflammation and insulin resistance.^21^^,^^22^ This duality positions IL-6 as a pivotal cytokine in exercise physiology, capable of mediating both beneficial and detrimental effects depending on the broader metabolic and inflammatory milieu.

The largest groupings of ethnicity proteins are modulators of immunity, inflammation and metabolism. The immunity and inflammatory proteins centred on a network of chemokines that were up-regulated in SAs compared to WEs (CCL11, CL13, CCL14, CCL26, CCL28). Conversely, CCXL14 was downregulated in SAs, acting as a central node. Although the specific chemokines identified in this study are novel, upregulation of CC chemokine ligands has previously been observed in SAs, supported by findings of higher concentrations, transmigration, and adhesion of monocytes compared with WEs.^23^ The downregulation of CXCL14 in SAs, validated in PROPELS, and its nodal positioning in the network analysis is notable. CXCL14, identified relatively recently, is thought to be involved in a broad range of biological actions centred on regulation of cell migration, immune surveillance and inflammation.^24^ It is proposed to act as tumour suppressor and is downregulated in many cancers.^24^ Recently, findings suggested CXCL14 may protect against insulin resistance by promoting the recruitment of non-inflammatory macrophages to adipose tissues, thereby supporting brown fat activity.^25^ Furthermore, levels of CXCL14 in plasma and expression in adipose tissue are abnormally downregulated in obesity and T2DM. It is, therefore, possible that the downregulation of CXCL14 contributes to the higher risk of T2DM observed at lower adiposity in SAs populations.^26^^,^^27^ However, further research is warranted to establish this association.

Metabolic proteins included fatty acid binding proteins and amylase. More specifically, FABP1 (mainly expressed in the liver) and FABP2 (small intestine) were upregulated in SAs compared to WEs. Animal models and epidemiological investigations suggest that downregulated FABP1 expression may suppress triglyceride accumulation in the liver, with therapeutic potential in the treatment of NAFLD.^28^^,^^29^ Given SAs have high levels of hepatic adiposity,^30^ FABP1 could have potential in elucidating the biological mechanisms underpinning these observations. Higher concentrations of AMY2A and AMY2B (expressed in the pancreas) are also likely to be relevant to ethnicity-related health disparities. AMY genes have been subject to strong pressures in recent human evolution and are reported to differ across ethnic groups.^31^^,^^32^ Amylase enzymes are responsible for the first phases of starch digestion and are hence important agents in carbohydrate metabolism and postprandial hyperglycemia. Pancreatic amylase is the most prevalent enzyme in acinar cells and is involved in the regulation of blood glucose levels, with alpha-amylase inhibitors developed as glucose lowering therapies.^31^ However, the role of pancreatic amylase in the aetiology of T2DM remains uncertain. The implications for ethnic health also warrant further investigation.

This is the first study to investigate the proteome of ethnicity, accelerometer-assessed physical activity change and their interaction. Furthermore, as the main analysis for this study was conducted following a discovery phase in a large independent cohort, the risk of type 1 error was reduced. However, the relatively small sample size, which was limited by practical implications (e.g., cost), increases the risk of a type 2 error. In particular, no proteins were selected and validated for a physical activity by ethnicity interaction, despite mounting clinical evidence that cardiometabolic responses to physical activity are modified by ethnicity which formed a key rationale for the current investigation.^8^, ^9^, ^10^ Larger samples with greater power to detect an interaction are therefore required to replicate and extend these findings. Indeed, a post hoc power calculation indicated that, with 30 South Asian and 28 White European individuals, the validation analysis had approximately 80% power to detect large effect sizes (standardised difference of >1) for the top 20 proteins identified. Therefore, the study was underpowered to identify more modest associations. It is also important to note that only a small, targeted part of the proteome was investigated through this analysis and a larger number of proteins (n = 3000) are available in the UK Biobank cohort. Accordingly, there may be important proteins that were not identified. While the inclusion of a discovery phase strengthens our analysis and adds to the robustness of the findings, this approach is also not without limitations. Notably, in UK Biobank, physical activity was self-reported and given the weak correlation between self-reported and objectively measured physical activity,^33^ it is possible that some proteins associated with objectively assessed physical activity were not identified at this stage. Similarly, differences in study design and population characteristics between the discovery and validation cohorts may have limited the generalisability across these stages. As a result, some proteins that were not selected in the discovery phase may have been relevant in the context of NDH. Nonetheless, cross–sectional associations may still reflect stable and biologically meaningful relationships that remain relevant over time, especially when the exposures in question (e.g., physical activity and ethnicity) are relatively constant or possess well-established biological effects. Therefore, testing whether the same proteins are associated with changes over time in an independent cohort provides an opportunity to validate these biological signals under different, but complementary, conditions. Moreover, despite the discrepancy in population characteristics, evidence suggests that the molecular and physiological responses to physical activity are broadly consistent across metabolic/chronic disease risk strata.^34^^,^^35^ In our case, the direction and magnitude of associations for IL-6 were consistent between the discovery and validation cohorts, suggesting that the protein–physical activity relationships we observed likely reflect shared biological mechanisms rather than population-specific effects. However, further mechanistic studies are warranted to substantiate these findings. Finally, although we analysed change in physical activity over a 48-month period, revealing a dynamic aspect of the proteins, the consequence of undertaking a post-hoc cohort analysis of a clinical trial involves the normal limitations of potential for unmeasured confounding or for reverse causation (for physical activity investigations).

A proteomics analysis of ethnicity and physical activity change identified mutually exclusive proteins associated for each trait, suggesting the inflammatory and cardiometabolic protein signatures of ethnicity and physical activity are largely distinct. As such, although physical activity may be beneficial for all, it may not work to normalise many of the ethnic specific differences linked to chronic disease. These findings, however, should be viewed as exploratory and hypothesis generating that warrant further research.

## Contributors

Conceptualisation (TY, JH); Data curation (TY, JH); Formal analysis (AT, AG, CR, THC); Methodology (TY, JH, AT, AG); Validation and verification (TY, JH); Visualisation (TY, JH, AG, AT, NCB, THC); Writing – original draft, and writing (TY, JH, AG, EJ); Review & editing (TY, JH, AT EJ, CLE, NCB, CR, DJLJ, THC, MJD, KK). All authors read and approved the final version of the manuscript. All authors also had full access to all the data in the study and accept responsibility to submit for publication.

## Data sharing statement

The data from the PROPELS trial that support the findings of this study are not publicly available due to a lack of specific patient consent and GDPR considerations. However, data can be made available from the corresponding author upon request for collaborative research purposes. The data from UK Biobank is available through https://www.ukbiobank.ac.uk/The UK Biobank data used in this analysis was through project number 33266.

## Declaration of interests

MJD has received grants or contracts from AstraZeneca, Boehringer Ingelheim, Novo Nordisk; consulting fees from Boehringer Ingelheim, Lilly, Novo Nordisk and Sanofi; payment or honoraria for lectures, presentations, speakers bureaus, manuscript writing or educational events from AstraZeneca, Boehringer Ingelheim, Novo Nordisk, Sanofi, Lilly; support for attending meetings and/or travel from Boehringer Ingelheim, Novo Nordisk, Lilly, Amgen, AstraZeneca, Biomea Fusion, Regeneron and Zealand Pharma; and participated on a Data Safety Monitoring Board or Advisory Board for Amgen, AstraZeneca, Biomea Fusion, Sanofi, Zealand Pharma, Carmot/Roche and Regeneron. KK has received grants or contracts from AstraZeneca, Boehringer Ingelheim, Lilly, MSD, Novo Nordisk, Sanofi, Servier, Oramed Pharmaceuticals, Roche, Daiichi-Sankyo, Applied Therapeutics; consulting fees from Amgen, AstraZeneca, Bristol Myers Squibb, Boehringer Ingelheim, Lilly, Novo Nordisk, Sanofi, Servier, Pfizer, Roche, Daiichi-Sankyo, Embecta and Nestle Health Science; and payment or honoraria for lectures, presentations, speakers bureaus, manuscript writing or educational events from Amgen, AstraZeneca, Bristol Myers Squibb, Boehringer Ingelheim, Lilly, Novo Nordisk, Sanofi, Servier, Pfizer, Roche, Daiichi-Sankyo, Embecta and Nestle Health Science. NB has received grants or contracts from Yakult Honsha and National Axial Spondyloarthritis Society; and consulting fees from Mars Wrigley. TY, THC, EJ, AG, AT, CE, CR, DJLJ and JH declare no conflicts of interest.

## Appendix A Supplementary data

Supplementary File 1 **Complete list of proteins and corresponding model results for the discovery phase (UK Biobank)**. Values are adjusted for age, sex, social deprivation smoking status, cancer, cardiovascular disease and diabetes; values reflect the normalised difference in protein levels in SAs *vs.* WEs. P-values are presented as nominal and after correction for multiple comparisons.

Supplementary File 2 **Proteins carried through from the discovery phase and corresponding model results for the validation phase (PROPELS)**. Values are adjusted for age, sex, randomisation sequence (control, Walking Away, Walking Away plus), and baseline steps (per day). Values reflect the normalised difference in protein levels. P-values are presented as nominal and after correction for multiple comparisons.

Supplementary File 3 **Gene Ontology (GO) enrichment analysis and pathway analysis using the Kyoto Encyclopaedia of Genes and Genomes (KEGG) for the validation phase (PROPELS)**.
