# Supplementary material for: Proteomics insights: exploring the inflammatory and metabolic signatures of ethnicity and change in physical activity in non-diabetic hyperglycaemia
Source: eBioMedicine. 2025 Nov 3;121:106006. doi: 10.1016/j.ebiom.2025.106006 (PMC12677085; doi:10.1016/j.ebiom.2025.106006)
Supplement: Supplementary Tables [file mmc4.docx]

**Table S1 Ethnicity-associated proteins in PROPELS**

| **Gene Name** | **Uniprot ID** | **Protein description** | **Ethnicity beta ^a^** | ***Nominal p-*value** | **95% CI** | **Ethnicity beta ^b^** | ***Adjusted* p-value *(Benjamini-Hochberg)*** | **95% CI** |
| --- | --- | --- | --- | --- | --- | --- | --- | --- |
| AMY2A | P04746 | Amylase Alpha 2A (Pancreatic) | 0.4813 | 0.0006 | (0.2186, 0.7441) | 0.4662 | 0.0016 | (0.2016, 0.7308) |
| AMY2B | P19961 | Alpha-amylase 2B | 0.4566 | 0.0013 | (0.1867, 0.7266) | 0.4417 | 0.0027 | (0.1697, 0.7137) |
| CCL11 | P51671 | C-C motif chemokine 11 | 0.3674 | 0.0018 | (0.1438, 0.5909) | 0.3566 | 0.0037 | (0.1306, 0.5827) |
| CCL13 | Q99616 | C-C motif chemokine 13 | 0.5592 | 0.0013 | (0.2303, 0.8882) | 0.5551 | 0.0029 | (0.2208, 0.8894) |
| CCL15 | Q16663 | C-C motif chemokine 15 | 0.5660 | 0.0007 | (0.2505, 0.8814) | 0.61106 | 0.0019 | (0.3059, 0.9162) |
| CCL26 | Q9Y258 | C-C motif chemokine 26 | 0.6093 | 0.0023 | (0.2281, 0.9904) | 0.6244 | 0.0040 | (0.2401, 1.0088) |
| CCL28 | Q9NRJ3 | C-C motif chemokine 28 | 0.7483 | 0.0003 | (0.3576, 1.1391) | 0.7185 | 0.0008 | (0.3271, 1.1098) |
| CD79B | P40259 | B-cell antigen receptor complex-associated protein beta chain | 0.4458 | 0.0014 | (0.1806, 0.7109) | 0.4486 | 0.0011 | (0.1789, 0.7183) |
| CXCL14 | O95715 | C-X-C motif chemokine 14 | -0.4801 | 0.0015 | (-0.7669, -0.1934) | -0.4621 | 0.0013 | (-0.7508, -0.1733) |
| FABP1 | P07148 | Fatty acid-binding protein 1 | 0.8324 | 0.0007 | (0.3716, 1.2933) | 0.9076 | 0.0019 | (0.4689, 1.3462) |
| FABP2 | P12104 | Fatty acid-binding protein 2 | 0.5474 | 0.0004 | (0.2593, 0.8355) | 0.5441 | 0.0021 | (0.2515, 0.8367) |
| FCRL6 | Q6DN72 | Fc receptor-like protein 6 | 0.6585 | 0.0004 | (0.3113, 1.0057) | 0.6577 | 0.0027 | (0.3043, 1.0111) |
| GHRL | Q9UBU3 | Growth hormone-releasing peptide | -0.9792 | 0.0001 | (-1.4431, -0.5154) | -1.0203 | 0.0003 | (-1.4834, -0.5573) |
| PRTN3 | P24158 | Leukocyte proteinase 3 | -0.5543 | 0.0010 | (-0.8724, -0.2362) | -0.5498 | 0.0011 | (-0.8724, -0.2272) |
| TFRC | P02786 | Transferrin receptor protein 1 | 0.4823 | 0.0032 | (0.1695, 0.7951) | 0.5126 | 0.0021 | (0.2028, 0.8224) |
| TNFRSF11A | Q9Y6Q6 | Tumor necrosis factor receptor superfamily member 11A | 0.42701 | 0.0002 | (0.2124, 0.6416) | 0.4790 | 0.0005 | (0.2953, 0.6628) |

^a^ Adjusted for age, sex, randomisation sequence and baseline steps; values represent normalized physical activity levels in South Asians (SAs) vs white Europeans (WEs).

^b^ As above and further adjusted for BMI.

**Table S2 Physical activity-associated proteins in PROPELS**

| **Gene Name** | **Uniprot ID** | **Protein description** | **Physical activity beta ^a^** | ***Nominal* p-value** | **95% CI** | **Physical activity beta ^b^** | ***Adjusted p-*value *(Benjamini-Hochberg)*** | **95% CI** |
| --- | --- | --- | --- | --- | --- | --- | --- | --- |
| IL6 | P05231 | Interleukin-6 | -0.1447 | <0.0001 | (-0.2136, -0.0756) | -0.1445 | 0.0002 | (-0.2136, -0.0756) |

^a^ Adjusted for age, sex, randomisation sequence and baseline steps; values represent normalized physical activity levels in increasers vs decreasers (per 12 months).

^b^ As above and further adjusted for body mass index (BMI).
